# Clinical characterization, genetic profiling, and immune infiltration of TOX in diffuse gliomas

**DOI:** 10.1186/s12967-020-02460-3

**Published:** 2020-08-06

**Authors:** Hao Zhang, Fan Fan, Yuanqiang Yu, Zeyu Wang, Fangkun Liu, Ziyu Dai, Liyang Zhang, Zhixiong Liu, Quan Cheng

**Affiliations:** 1 Department of Neurosurgery, Xiangya Hospital, Central South University, Changsha, 410008 Hunan People’s Republic of China; 2 Department of Clinical Pharmacology, Xiangya Hospital, Central South University, Changsha, 410008 Hunan People’s Republic of China; 3 Center for Medical Genetics and Hunan Provincial Key Laboratory of Medical Genetics, School of Life Sciences, Central South University, Changsha, China; 4 National Clinical Research Center for Geriatric Disorders, Xiangya Hospital, Central South University, Changsha, Hunan China; 5 Department of Medicine, The University of Oklahoma Health Sciences Center, Oklahoma City, OK 73104 USA; 6 Clinical Diagnosis and Therapeutic Center of Glioma, Xiangya Hospital, Central South University, Changsha, 410078 Hunan People’s Republic of China

**Keywords:** Glioma, TOX, Inflammatory activity, Immune response, Prognosis

## Abstract

**Background:**

Immunotherapies targeting glioblastoma (GBM) have led to significant improvements in patient outcomes. TOX is closely associated with the immune environment surrounding tumors, but its role in gliomas is not fully understood.

**Methods:**

Using data from The Cancer Genome Atlas (TCGA) and the Chinese Glioma Genome Atlas (CGGA), we analyzed the transcriptomes of 1691 WHO grade I-IV human glioma samples. The R language was used to perform most of the statistical analyses. Somatic mutations and somatic copy number variation (CNV) were analyzed using GISTIC 2.0.

**Results:**

TOX was down-regulated in malignant gliomas compared to low grade gliomas, and upregulated in the proneural and IDH mutant subtypes of GBM. TOX^low^ tumours are associated with the loss of PTEN and amplification of EGFR, while TOX^high^ tumours harbor frequent mutations in IDH1 (91%). TOX was highly expressed in leading edge regions of tumours. Gene ontology and pathway analyses demonstrated that TOX was enriched in multiple immune related processes including lymphocyte migration in GBM. Finally, TOX had a negative association with the infiltration of several immune cell types in the tumour microenvironment.

**Conclusion:**

TOX has the potential to be a new prognostic marker for GBM.

## Background

Gliomas continue to be the most common and devastating primary brain tumor. Despite multiple conventional therapies, including radiotherapy with adjuvant temozolomide chemotherapy after resection [1, 2], patients with LGG (low grade glioma) have a median overall survival (OS) of 8–10 years, while patients with GBM (glioblastoma multi-form) have a dismal OS of less than 15 months [3, 4]. Therefore, new therapeutic approaches are desperately needed.

Under normal physiological circumstances, immune checkpoints have proven to be responsible for the immune system self-tolerance [5, 6]. Recently, strategies eliciting immune responses against tumors have demonstrated breakthroughs in several cancer types [7–9]. One previous study demonstrated that tumor microenvironment, including infiltrating immune cells, play critical roles in supporting glioma progression [10]. Subsequent glioma immunotherapy research portends a promising future for the treatment of glioma patients [11, 12]. Emerging evidence demonstrates that immune checkpoints act as a crucial mediator of GBM through resident immune components [13, 14]. For example, B7‐H3 (CD276) is an immune checkpoint mainly expressed on T cells and thought to regulate the T cell‐mediated immune response. High expression of CD276 is associated with the extent of tumor malignancy [15]. IDO1, another immune checkpoint expressed in T cells, can promote a regulatory phenotype in both T cells and dendritic cells through its activity, effectively facilitating tumor immune escape [16, 17]. However, despite multiple studies, the intricate interactions between gliomas and the immune system remain to be fully elucidated [18].

Thymocyte selection-associated high mobility group box (TOX), a member of a conserved DNA-binding protein family, is closely associated with the regulation of the development of several immune-cell lineages including CD4 T cells, natural killer cells, and lymphocytes [19, 20]. TOX expression is frequently up-regulated in diverse types of cancer including breast cancer, lung cancer, cutaneous lymphoma, gastric cancer, leukemia, and central neural lymphoma. Multiple studies have demonstrated that the overregulation of TOX is associated with tumor progression [21]. Deregulation of TOX expression in cancer can be roughly attributed to two mechanisms: genetic alteration [22, 23] and epigenetic events [24]. While TOX is proven to be a critical regulator in the differentiation and maturation of the immune system, little is known about the immune-related roles of other three TOX protein family members. TOX2 was reported to play potential roles in reproductive organogenesis [25] and cancer [24]. TOX3 is involved in the regulation of neuron [26] and oligodendrocyte [27] survival, while it also plays multiple roles in breast cancer [28]. TOX4, a platinated-DNA interacting protein, interacts with a complex, controlling cell cycle kinetics and chromatin structure [29].

To date, TOX expression has not been fully characterized in gliomas. In this study, we investigated the role of TOX expression, aiming to comprehensively delineate its molecular and clinical patterns. To explore its clinical relevance with LGG and GBM, we mined data from the TCGA dataset and our findings were further validated using the CGGA dataset. This is the first integrative study characterizing TOX expression in LGG and GBM molecularly and clinically. A better understanding of TOX features and expression in gliomas may further promote research into associated therapies.

## Materials and methods

### Data collection

This study was ethically approved by Xiangya Hospital, Central South University. We collected TOX data from LGG and GBM samples in the TCGA and CGGA databases. 672 samples from TCGA were downloaded from UCSC Xena (https://xenabrowser.net/). 1013 samples were downloaded from the CGGA website (http://www.cgga.org.cn/). CGGA samples included those combined from mRNAseq_693 (batch 1) and mRNAseq_325 (batch 2). Fragments per kilobase million (FPKM) values were transformed into transcripts per kilobase million (TPM) values, which are more comparable between samples [30]. R package sva was then used to reduce the computational batch effect. RNA-seq data in regard to specific tumor anatomic structure in GBM was downloaded from the Ivy Glioblastoma Atlas Project (http://glioblastoma.alleninstitute.org/). 8295 normal samples from both TCGA and GTEX (http://commonfund.nih.gov/GTEx/) databases were used for comparisons of tumor and normal tissue.

### Biological function and gene set variation analysis

The patients were divided into TOX^high^ and TOX^low^ groups according to the median expression value of TOX. Correlation analysis of TOX was performed using gene expression profiles from the TCGA and CGGA datasets using the R language (https://www.r-project.org/). Somatic mutations and somatic copy number alternations (CNAs) which correspond to the cases with RNA-seq data, were downloaded from TCGA database. GISTIC analysis was adopted to determine the genomic event enrichment. CNAs associated with TOX expression and the threshold copy number at alteration peaks were obtained from the GISTIC 2.0 analysis (https://gatkforums.broadinstitute.org). The gene sets variation analysis (GSVA) package was used to analyze the differential expression in GO terms of immune related processes and immune cell lineages from TCGA and CGGA samples. Correlation analysis was performed by the expression values of risk score and GO term, and items with p < 0.05 and high correlation coefficient were selected. After Spearman correlation analysis, heatmaps were used to exhibit the gene expression pattern in the two most TOX-correlated GO pathways.

### Statistical analysis

Spearman correlation analysis was used to evaluate the correlations between continuous variables. The survival probability was determined using Kaplan–Meier survival curves. The Student t-test, χ2 test, or Pearson’s Chi squared test were used to determine the expression levels of TOX with regard to pathological characteristics. The Pearson correlation was applied for evaluating the linear relationship between gene expression levels. The survival package in R was used for Cox regression analysis. All statistical analyses were performed using R version 3.4.1. P-values < 0.05 were considered to be statistically significant and all tests were two-sided.

## Result

### TOX expression is decreased in malignant gliomas

**Fig. 1:**
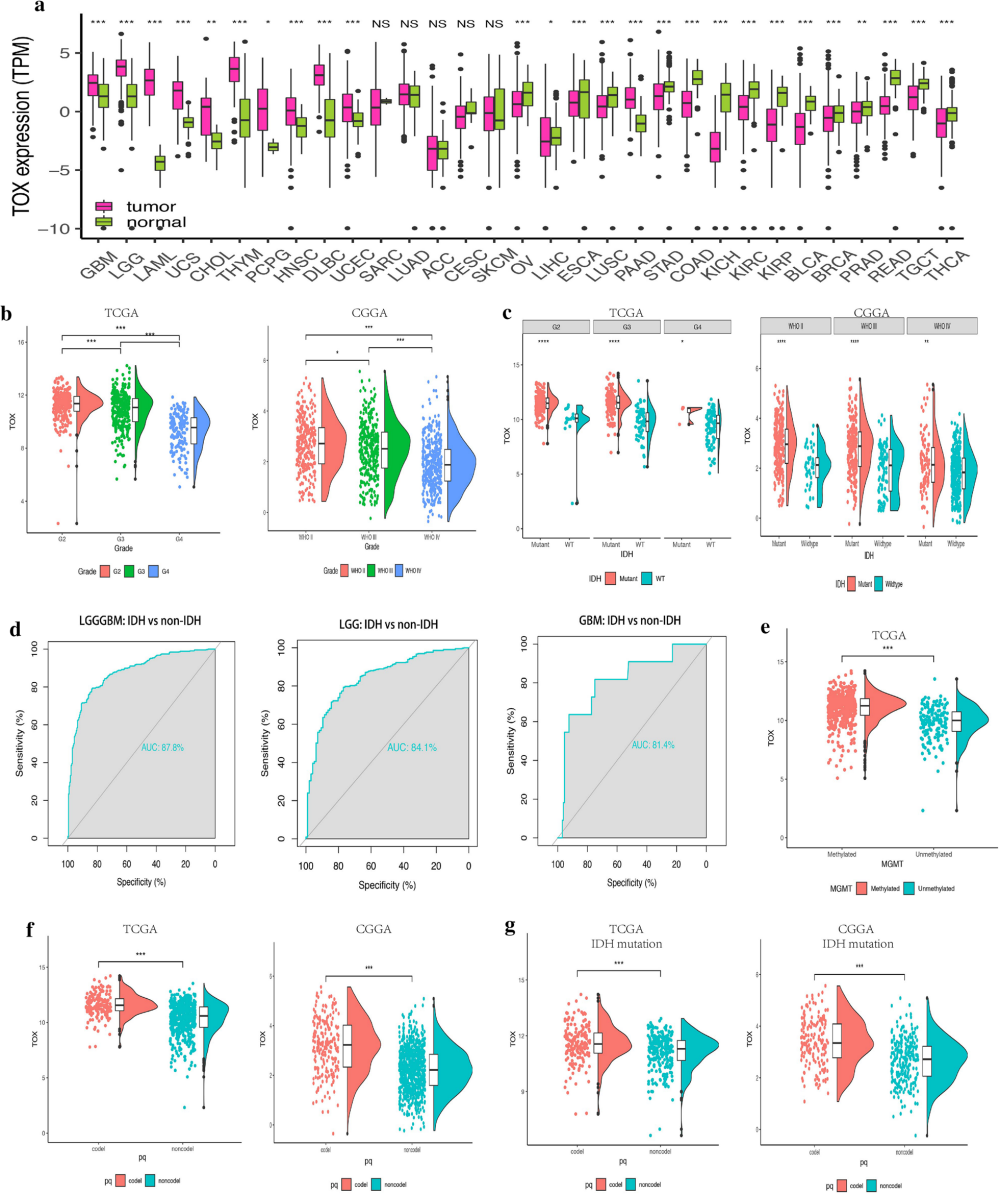
TOX expression is upregulated in malignant gliomas. **a** Analysis of TOX mRNA levels (log2) in different tumours from TCGA. **b** Analysis of TOX mRNA levels in WHO grade II-IV gliomas from TCGA and CGGA. **c** TOX expression is upregulated in IDH mutant gliomas compared with the IDH wild-type gliomas from TCGA and CGGA. **d** Receiver operating characteristic (ROC) curves assessing the sensitivity and specificity of TOX expression as a predictor of IDH mutation in gliomas from TCGA. **e** TOX expression is upregulated in the MGMT promoter methylated gliomas from TCGA. **f** TOX expression is upregulated in 1p/19q codeletion gliomas compared with 1p/19q non-codeletion gliomas from TCGA and CGGA. **g** TOX expression is upregulated in 1p/19q codeletion as well as IDH mutant gliomas from TCGA and CGGA

We obtained data from publicly available data-bases (TCGA, n = 674; CGGA, n = 1017) to evaluate the mRNA expression levels of TOX in WHO grade I-IV gliomas. First, we evaluated TOX levels in various common cancer types including gliomas (Fig. 1a). Compared to normal brain tissue, tumor samples demonstrated significantly up-regulated TOX expression, suggesting an association with glioma development. TOX was significantly elevated in low-grade glioma (LGG) samples compared with GBM samples (Fig. 1b). Interestingly, TOX had the highest expression in WHO grade II samples in both the TCGA and CGGA datasets (Fig. 1b).

**Fig. 2:**
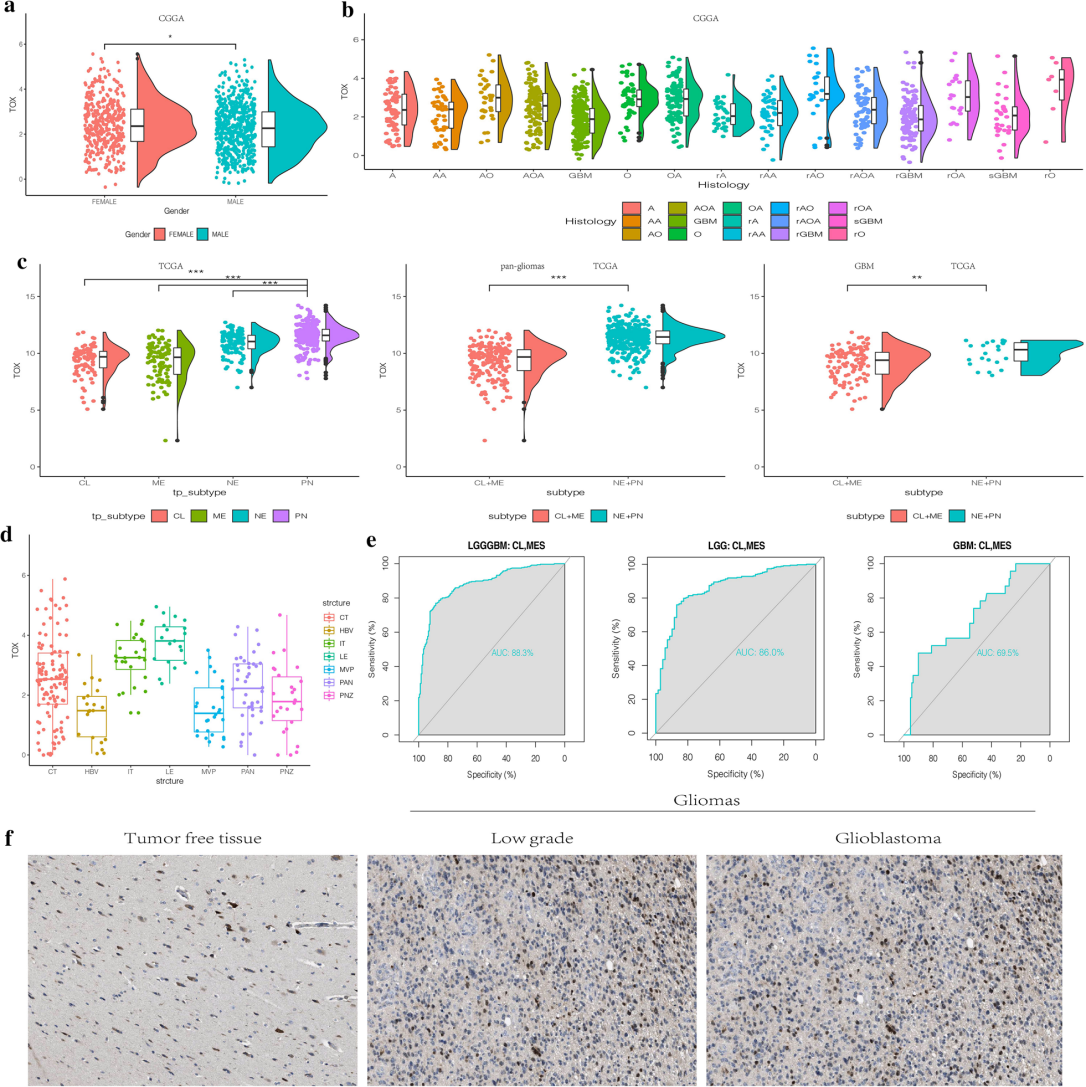
**a** TOX expression is upregulated in female patients with gliomas from CGGA. **b** The expression levels of TOX based on the histopathologic classification from CGGA. A, low-grade astrocytoma; AA, anaplastic astrocytoma; AO, anaplastic oligodendroglioma; GBM, glioblastoma; O, oligodendroglioma; rA, recurrent low-grade astrocytoma; rAA, recurrent anaplastic astrocytoma; rGBM, recurrent glioblastoma; rO, recurrent oligodendroglioma; sGBM, sensitive glioblastoma; AOA, anaplastic oligoastrocytoma; OA, oligoastrocytoma. **c** The TOX expression pattern in the TCGA molecular subtype in pan-glioma analysis and GBM samples. **d** TOX expression is detected in different anatomic locations for GBM in the IVY GBM database. LE (Leading Edge), IT (Infiltrating Tumour), CT (Cellular Tumour), PAN (Pseudopalisading Cells Around Necrosis), PNZ (Perinecrotic Zone), MVP (Microvascular Proliferation), and HBV (Hyperplastic Blood Vessels). **e** ROC curves predict that TOX is a biomarker of classical and mesenchymal subtype glioma. **f** TOX is more highly expressed in LGG than in GBM at the protein level

Isocitrate dehydrogenase (IDH) mutation, which is associated with better clinical outcomes, has a tight association with a high expression level of TOX (Fig. 1c). Furthermore, in WHO grade II glioma samples, the IDH mutant tumors had the highest expression of TOX in both TCGA and CGGA cohorts (Fig. 1c). The ROC curve further suggested that TOX could be a valuable predictor for IDH mutation across glioma types, in LGG cases, and in GBM cases respectively (AUC value = 0.878, P < 0.001; value = 0.841, P < 0.001; value = 0.814, P < 0.001, respectively Fig. 1d). In addition, higher expression of TOX was related to MGMT promoter methylation in the TCGA cohort (Fig. 1e). Additionally, TOX was up-regulated with 1p/19q codeletion in pan-glioma analysis in both TCGA and CGGA cohorts (Fig. 1f). Notably, in LGG samples, IDH mutation together with 1p/19q codeletion is related to higher expression of TOX in both TCGA and CGGA cohorts (Fig. 1g). In the CGGA cohort, females had relatively higher expression levels of TOX (Fig. 2a). The different expression levels of TOX in glioma in regard to histology is shown in Fig. 2b.

### Molecular characteristics of TOX in gliomas

The molecular categorization of human gliomas has four distinct sub-classes: mesenchymal (MES), classical (CL), neural (NE), and proneural (PN). MES and CL subtypes are related to more aggressive behavior of gliomas and more dismal clinical outcome of patients compared with PN or NE subtypes [31, 32]. Therefore, we subsequently analyzed the expression level of TOX among these four molecular subtypes on the basis of VERHAAK_2010 classification scheme [33]. In the TCGA dataset, lower TOX expression was seen in MES and CL subtypes of GBM compared to NE and PN subtypes, while the distinction was conspicuous in pan-glioma analysis (Fig. 2c). The ROC curve further indicated that TOX might serve as a predictor for CL and MES subtypes in pan-gliomas analysis, LGG alone, and GBM alone (AUC value = 0.883, P < 0.001; value = 0.860, P < 0.001; value = 0.695, P < 0.001, respectively Fig. 2e). Moreover, the highest TOX expression was seen in the PN molecular subtype in GBM samples (Fig. 2c).

We next evaluated the intra-tumour distribution of TOX in GBM samples. Based on the IVY GBM database, the analysis of RNA sequencing data revealed the high expression of TOX in cellular tumour, leading edge, and infiltrating tumour (Fig. 2d). To further confirm the upregulation of TOX expression at the protein level, we downloaded the results of immunohistochemistry (IHC) staining for TOX from the The Human Protein Atlas (https://www.proteinatlas.org) (Fig. 2f). TOX demonstrated higher expression in LGG and GBM compared to normal brain tissue. The expression of TOX was also higher in LGG than GBM, which is consistent with our previous results.

### TOX expression predicts better survival probability in glioma

**Fig. 3:**
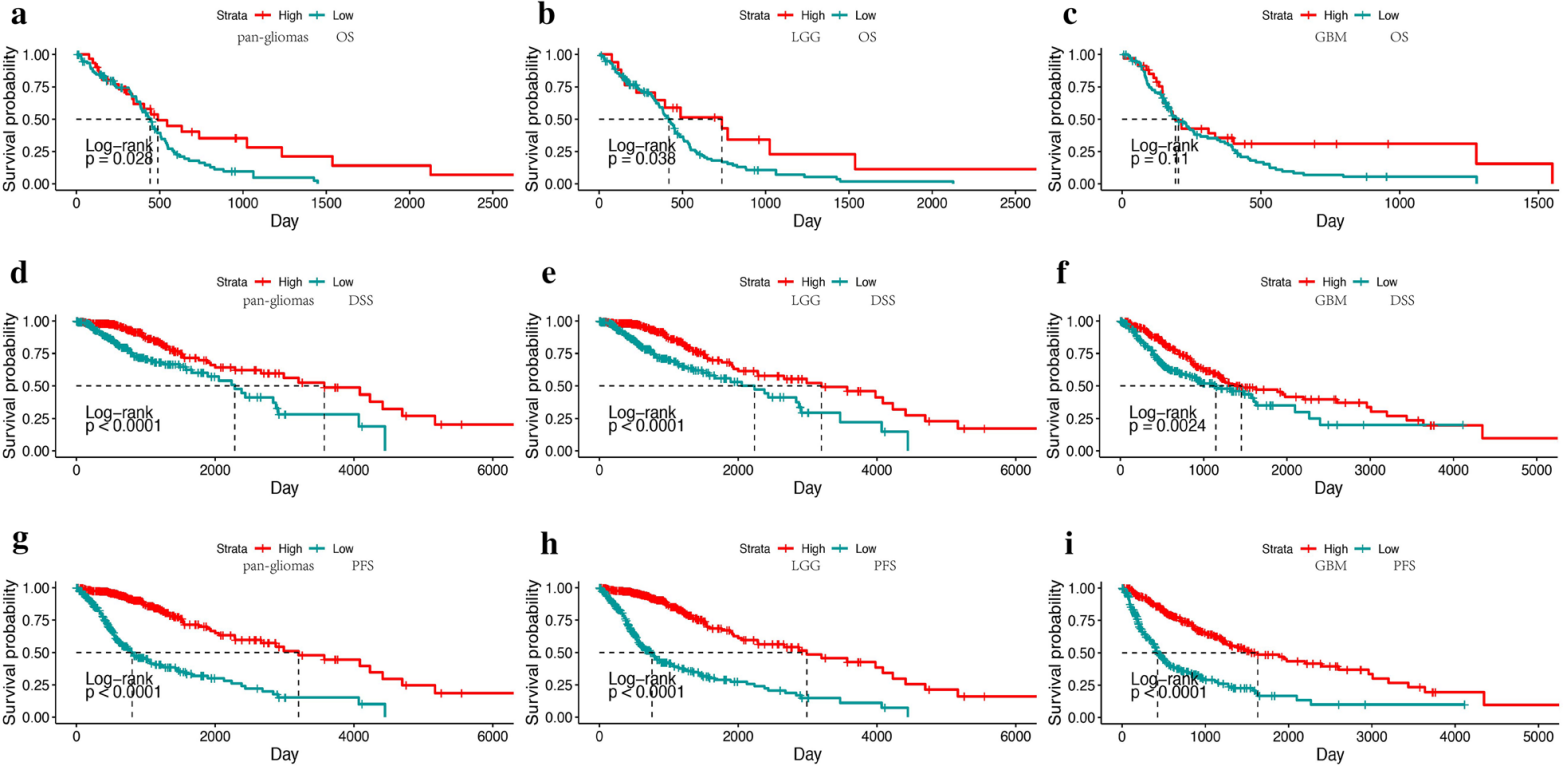
TOX expression predicts better survival in glioma patients. Kaplan–Meier analysis of overall survival (OS), disease specific survival (DSS) and progressive free survival (PFS) based on high vs low expression of TOX in pan-glioma analysis, LGG alone, and GBM alone in the TCGA dataset. The median value of TOX expression was used as the cut-off value. P-values were obtained from the log-rank test

We further investigated the prognostic value of TOX in human gliomas. Based on the calculated median values of TOX expression in gliomas, we generated Kaplan–Meier survival curves. In TCGA GBM dataset, TOX^high^ patients exhibited significantly longer overall survival (OS), disease specific survival (DSS), and progression free survival (PFS) compared with TOX^low^ patients (P < 0.05, respectively; Fig. 3a–c). In addition, in TCGA LGG datasets, TOX^high^ patients exhibited significantly longer overall survival (OS), disease specific survival (DSS), and progressive free survival (PFS) compared with TOX^low^ patients (P < 0.001, respectively; Fig. 3d–f). This result was further confirmed in pan-glioma analysis (P < 0.001, respectively; Fig. 3g–i). In the CGGA dataset, TOX^high^ patients had longer OS in pan-glioma, LGG, and GBM analyses (P < 0.001, P < 0.001 and P < 0.05 respectively; Additional file 1: Fig. S1A–C). Furthermore, Cox regression analysis was performed to explore the clinical prognostic value of TOX in gliomas. In univariate analysis, TOX, WHO Grade, age at diagnosis, 1p19q codeletion, and IDH mutation were significantly related to OS in both TCGA and CGGA databases (Tables 1, 2). In multivariate analysis, TOX also proved to be a valuable predictor in both cohorts. These results reveal that TOX might serve as an independent predictor of prognosis in glioma patients.

**Table 1:** Univariate and multivariate cox analyses in gliomas in CGGA

Factor	CGGA RNA-seq set					
	Univariate			Multivariate		
	P	HR	95% CI	P	HR	95% CI
TOX						
High vs. low	< 0.001	2.34	1.95–2.82	< 0.001	1.44	1.18–1.76
Age						
Increasing years	< 0.001	1.03	1.02–1.04	0.044	1.01	1.00–1.02
Gender						
Male vs. female	0.823	1.02	0.85–1.22	0.518	0.98	0.82–1.17
WHO grade						
Grade III vs. II	< 0.001	3.01	2.26–4.00	< 0.001	2.90	2.17–3.86
Grade IV vs. II	< 0.001	8.53	6.40–11.01	< 0.001	5.22	3.88–7.02
1p19q status						
Codel vs. non-codel	< 0.001	4.41	3.26–5.97	< 0.001	2.56	1.85–3.55
IDH status						
Mutation vs. wild-type	< 0.001	3.12	2.59–3.75	0.023	1.23	0.99–1.54

**Table 2:** Univariate and multivariate cox analyses in gliomas in TCGA

Factor	TCGA RNA-seq set					
	Univariate			Multivariate		
	P	HR	95% CI	P	HR	95% CI
TOX						
High vs. low	< 0.001	4.32	3.24–5.77	0.044	1.47	1.01–2.14
Age						
Increasing years	< 0.001	1.06	1.05–1.07	< 0.001	1.03	1.02–1.04
Gender						
Male vs. Female	0.084	1.26	0.97–1.63	0.229	1.20	0.90–1.56
WHO grade						
Grade III vs. II	< 0.001	3.34	2.28–4.89	< 0.001	2.20	1.48–3.26
Grade IV vs. II	< 0.001	17.95	12.11–26.59	< 0.001	3.64	2.23–5.95
1p19q status						
Codel vs. Non-codel	< 0.001	4.23	2.75–6.51	0.012	1.88	1.15–3.10
IDH status						
Mutation vs. wild-type	< 0.001	8.92	6.76–11.75	< 0.001	2.27	1.47–3.51

### The association between TOX expression levels and distinct genomic alterations

**Fig. 4:**
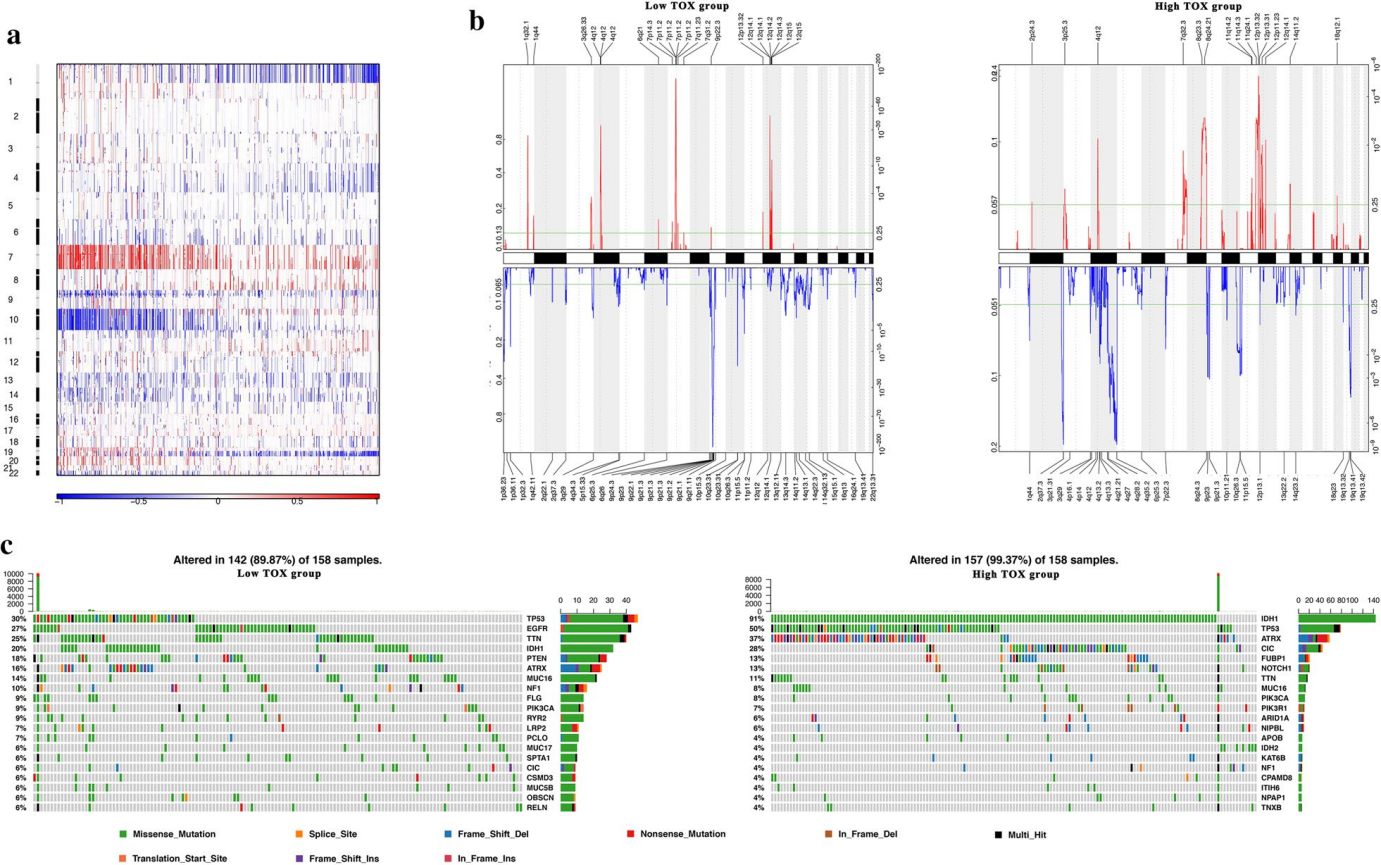
Distinct genomic profiles are associated with TOX expression. **a** The overall CNA profile in order of increasing TOX expression. Number 1 to 22 represents 22 human chromosomes. **b** GISTIC 2.0 amplifications and deletions in gliomas with low and high TOX expression. Chromosomal locations of peaks of significantly recurring focal amplification (red) and deletions (blue) are presented. **c** Differential somatic mutations are detected in gliomas with low and high TOX expression

We next performed somatic mutation analysis and copy number variation (CNV) using the TCGA dataset to determine whether TOX expression levels were associated with specific genomic characteristics. By comparing TOX^low^ (n = 158) and the TOX^high^ (n = 158) clusters (Fig. 4c), we obtained an overall CNV profile. Chromosome 7 amplification and chromosome 10 deletion, the two most common genomic events in GBM, were frequently associated with the TOX^low^ cluster (Fig. 4a). The genomic hallmark of oligodendroglioma, deletion of 1p and 19q, was more frequently occurring in the TOX^high^ cluster (Fig. 4a).

We next identified 43 and 61 genomic events enriched in either the TOX^high^ or TOX^low^ group using GSITIC analysis (Fig. 4b). In TOX^low^ samples, oncogenic driver genes including PIK3C2B (1q32.1), PDGFRA (4q12), EGFR (7p11.2), and CDK4 (12q14.1) were frequently amplified genomic regions. Meanwhile, frequently deleted genomic regions included tumour suppressor genes such as PARK7 (1p36.23), CDKN2A (9p21.3), and PTEN (10q23.3). In TOX^high^ samples, 8q23.3 and 12p32.32 were two significant amplified peaks, while significant deletion peaks were detected in 2q37.3, 4q35.2, 9p21.3, 11p15.5, and 19q13.43. Notably, a 4q12 peak was detected in both TOX^high^ and TOX^low^ samples. However, the G score in TOX^high^ samples was notably higher than that of TOX^low^ samples. Based on TOX expression levels, the somatic mutation profiles revealed that mutations in IDH1 (91%), CIC (28%), and ATRX (37%) were significantly enriched in GBM samples with high TOX expression (Fig. 4c). In addition, frequently observed mutations to EGFR (27%), IDH1 (20%), PTEN (18%), and MUC16 (16%) were present in gliomas with low TOX expression (n = 158; Fig. 4c).

### TOX is involved in complex immune processes of the tumor

**Fig. 5:**
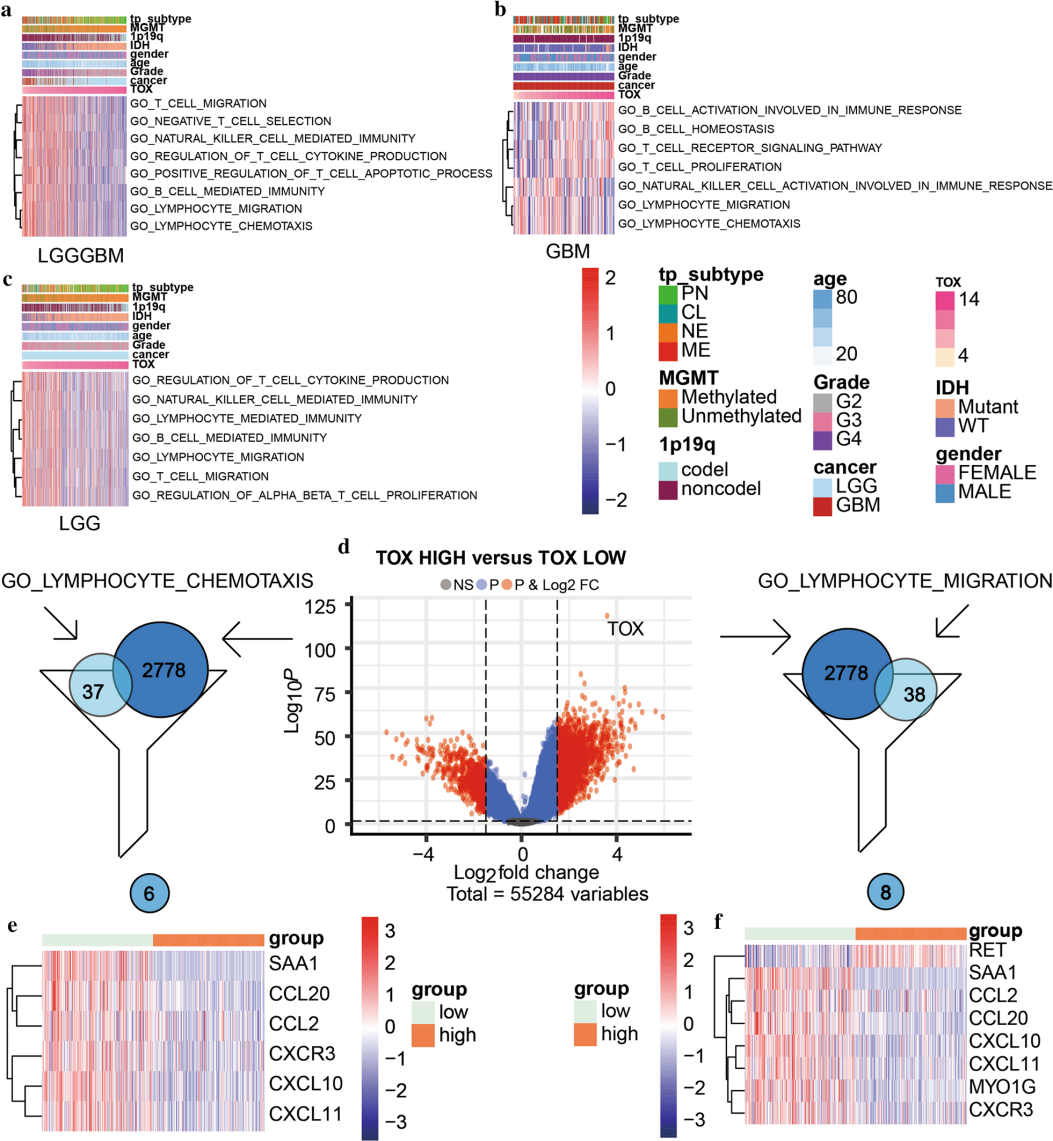
TOX related immune processes in pan-glioma analysis (**a**), LGG (**b**) and GBM (**c**) patients in the TCGA dataset. **d** Volcano plot for differentially expressed genes (DEGs). **e** Genes present in both the lymphocyte chemotaxis gene set and DEGs. **f** Genes present in both the lymphocyte migration gene set and DEGs

We further investigated the potential immune-related functions of TOX in glioma using GSVA analysis in TCGA dataset. In GBM alone, we found that TOX was positively associated with B cell activation, T cell receptor signaling pathway, B cell homeostasis, and T cell proliferation. In contrast, TOX had negative association with lymphocyte migration, natural killer cell activation, and lymphocyte chemotaxis (Fig. 5b). In pan-glioma analysis, TOX had a negative association with T cell migration, negative T cell selection, natural killer cell mediated immunity, regulation of T cell cytokine production, positive regulation of T cell apoptotic process, B cell mediated immunity, lymphocyte migration, and lymphocyte chemotaxis (Fig. 5a). In LGG alone, TOX was negatively related to T cell migration, lymphocyte migration, regulation of T cell cytokine production, lymphocyte mediated immunity, and regulation of αβ T cell proliferation. Similar results were seen in the CGGA dataset (Fig. 5c).

A previous study has demonstrated that TOX is essential in the development and differentiation of innate lymphoid cells [34]. Consequently, we paid special attention to two pathways mentioned above: lymphocyte migration and lymphocyte chemotaxis. With a threshold set at logFC > 2 and adjusted P-value ≦ 0.01, a total number of 2778 differentially expressed genes (DEGs) were detected between samples with high expression of TOX and low expression of TOX (Fig. 5d). For lymphocyte migration, eight genes were found present in both DEGs and lymphocyte migration gene sets. SAA1, CXCL11, CXCL10, CCL2, CCL20, CXCR3, and MYO1G were related with high expression of TOX, whereas RET was related with low TOX expression. For lymphocyte chemotaxis, expression of TOX was negatively related to SAA1, CXCL11, CXCL10, CCL2, CCL20, CXCR3 (Fig. 5e, f). These findings demonstrate a link between TOX expression and immune processes in glioma.

### TOX is irrelevant to inflammatory activities

**Fig. 6:**
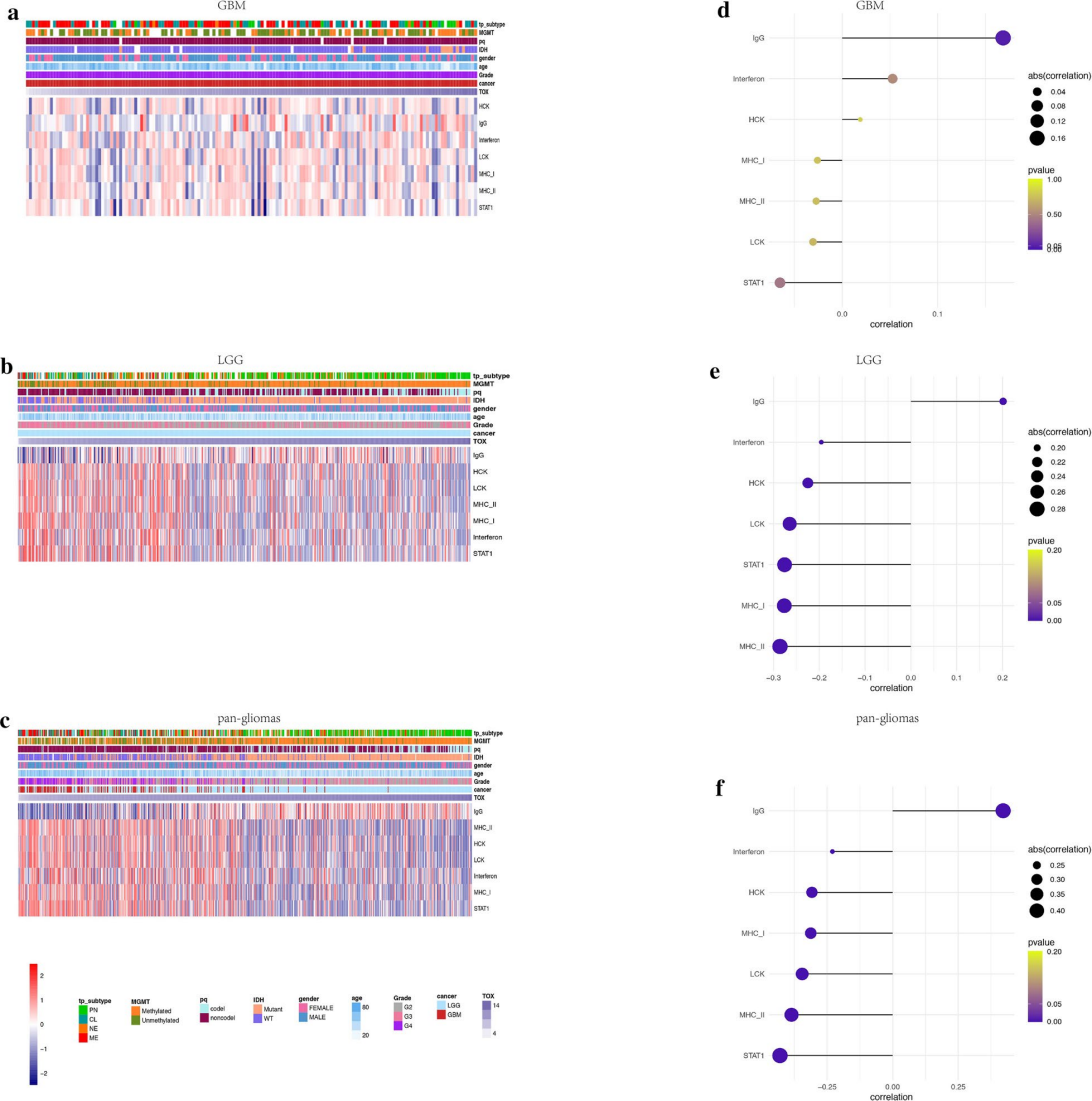
Heatmaps illustrating TOX related inflammatory activities in GBM (**a**), LGG (**b**) and pan-glioma analysis (**c**) in the TCGA dataset, respectively. Expression values are z-transformed and are colored red for high expression and blue for low expression, as illustrated in the scale bar. Correlation-grams illustrate P values for analysis between TOX and inflammatory metagenes in GBM (**d**), LGG (**e**) and pan-glioma analysis (**f**) in the TCGA dataset, respectively

We examined the association between TOX associated immune genes and various molecules related to inflammatory activity in both TCGA and CGGA datasets. TOX expression was negatively associated with inflammatory activity signatures including HCK, LCK, MHC-I, MHC-II, STAT1, and interferon metagenes, but positively associated with the IgG metagene in pan-glioma analysis, LGG alone, and GBM alone (Fig. 6a–c; Fig. Additional file 1: S1D–G). These results indicate that TOX is not involved in signaling transduction of T cell activation, macrophage activation, or antigen presenting cells (APCs). However, TOX might interact with B lymphocytes in the processes of immune-activation and subsequent glioma suppression.

### TOX and immune cells are tightly associated in the tumour microenvironment

**Fig. 7:**
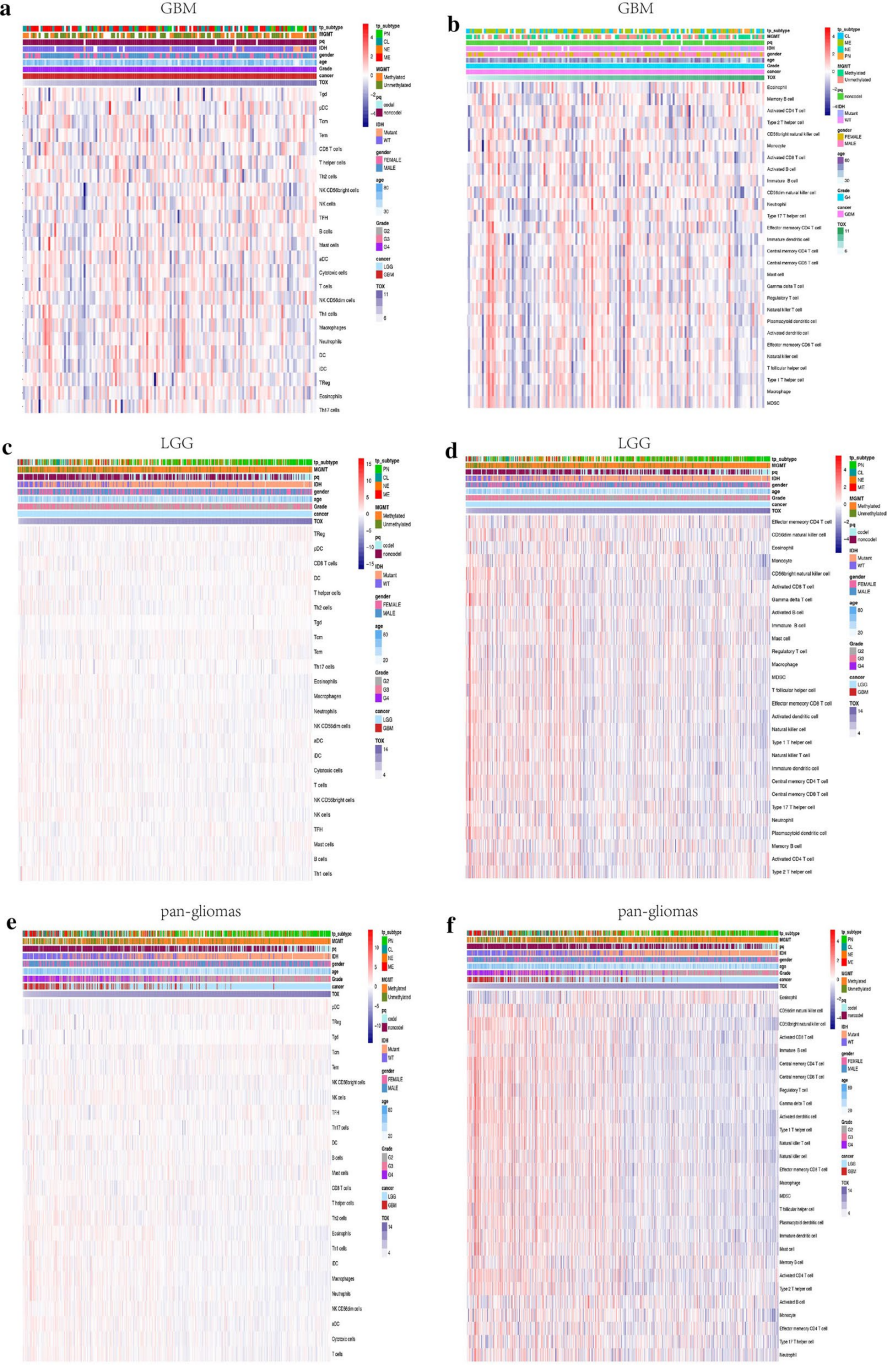
TOX is associated with immune cells in the tumour microenvironment. Heatmaps illustrating the relationship between TOX and 24 immune cell populations based on TCGA GBM (**a**), LGG (**c**) and pan-glioma analysis data (**e**), respectively. Heatmaps illustrating the relationship between TOX and 28 immune cell populations based on TCGA GBM (**b**), LGG (**d**) and pan-glioma analysis data (**f**), respectively. The z-transformed expression values are colored red for high expression and blue for low expression, as indicated in the scale bar

We further examined the significance of increased TOX in immune-related microenvironment in gliomas via GSVA analysis. We identified the immune cell types in the microenvironment of gliomas to see if they are influenced by TOX and to evaluate its presumed role in the interaction between gliomas and immune cells. We first investigated the relationship between TOX and 28-immune cell populations using cell type gene set variation analysis [35]. In both TCGA and CGGA cohorts, we found that TOX was positively associated with eosinophils in pan-glioma analysis, whereas multiple immune cell types with infiltration characteristics including macrophages, monocytes, CD4+ TEM, CD8+ T effector memory cells (TEM), neutrophils, myeloid-derived suppressor cells (MDSC), and natural killer (NK) cells were negatively associated with TOX in pan-glioma analysis and in LGG analysis (Fig. 7d, f; Additional file 2: Fig. S2, Additional file 3: Fig. S3). For GBM samples, DCs, MDSC, macrophages, mast cells, NK cells, CD8+ TEM, and CD4+ TEM were found to be negatively associated with TOX (Fig. 7b; Additional file 2: Fig. S2, Additional file 3: Fig. S3). We further validated these results in a 24-immune cell lineage analysis, confirming the rejection of multiple immune cell types [36] in TOX^high^ glioma samples. In the 24-immune cell lineage analysis, neutrophils, eosinophils, macrophages, NK cells, and DCs were negatively associated with TOX. TFH (follicular helper cells) and tumor growth delay (TGD) were positively associated with TOX in the pan-glioma analysis and the LGG group (Fig. 7c, e; Additional file 2: Fig. S2, Additional file 3: Fig. S3), while TFH and B cells were positively associated with TOX, and macrophages and DCs were negatively associated with TOX in GBM samples (Fig. 7a; Additional file 2: Fig. S2, Additional file 3: Fig. S3). Altogether, our data reveal that high expression of TOX is associated with reduced infiltration of immune cells in the microenvironment of gliomas.

### TOX is synergistic with other immune checkpoint members

**Fig. 8:**
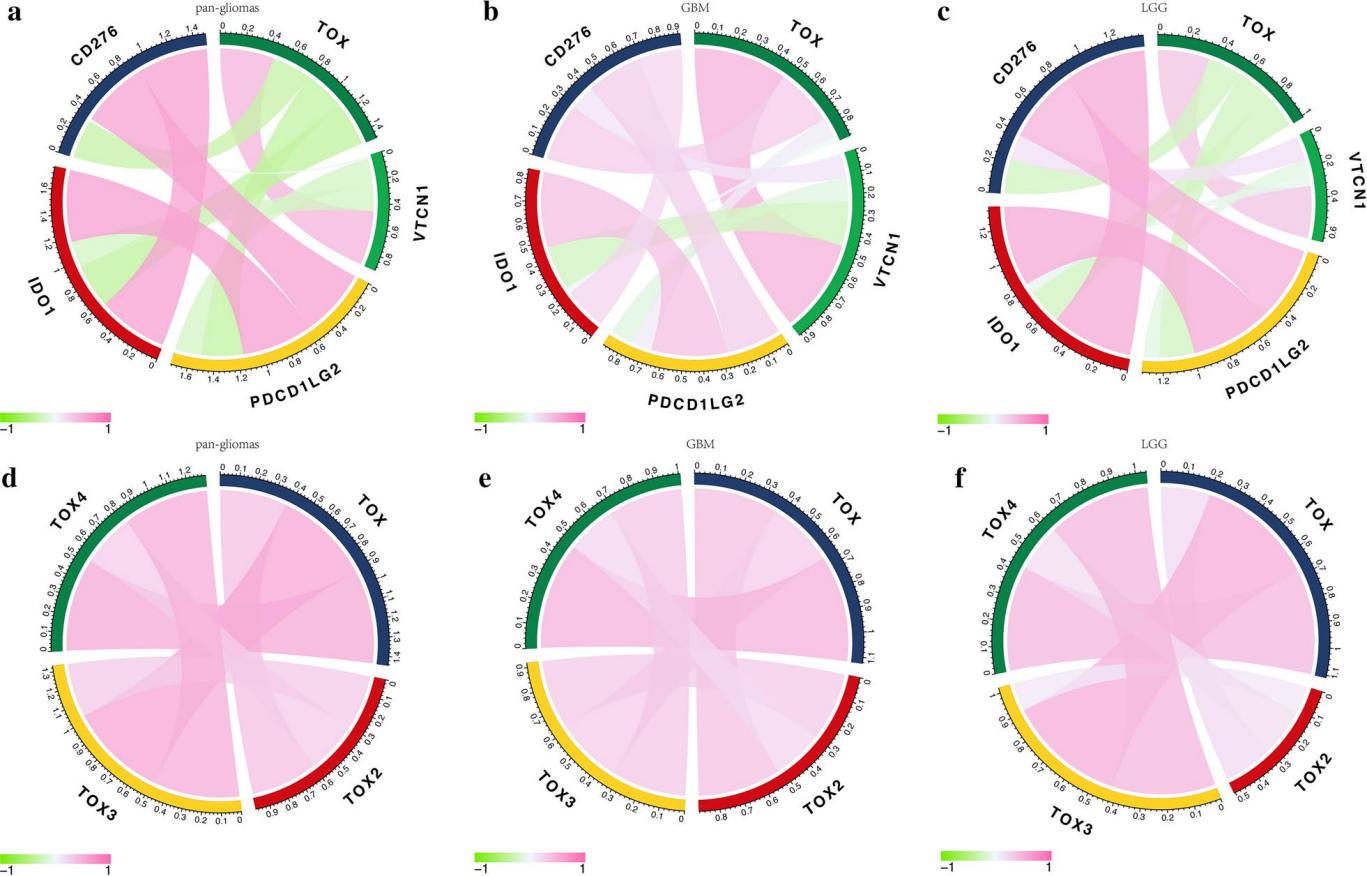
TOX is correlated with classic immune checkpoint molecules in gliomas. Correlation of TOX and immune checkpoint members in pan-glioma analysis (**a**), GBM (**b**) and LGG (**c**) samples in TCGA. Correlation of TOX and other TOX protein family members in pan-glioma analysis (**d**), GBM (**e**) and LGG (**f**) samples in TCGA

Given that the immune checkpoint molecules vitally regulate immune processes, we assessed the correlation between TOX and several crucial immune checkpoints in glioma samples. TOX was strongly correlated with CD276, IDO1, PDCD1LG2 (PD-L2), and VTCN1 in pan-glioma analysis and GBM alone in both TCGA and CGGA cohorts (Fig. 8a, b; Additional file 4: Fig. S4). The correlation was significantly better in LGG samples alone (Fig. 8c; Additional file 4: Fig. S4). The analysis of TOX protein family members showed coexpression of TOX, TOX2, TOX3, and TOX4 in pan-glioma analysis, LGG alone, and GBM alone (Fig. 8d–f; Additional file 4: Fig. S4).

## Discussion

After many years of research, gliomas, especially GBM, remain the most devastating brain tumors with dismal outcomes. Strategies eliciting an immune response against the tumor have led to breakthroughs in preclinical and clinical trials in many malignant tumors. TOX together with other classical immune checkpoints including PD1 and CD270 are closely related to the development of several immune-relevant cell subsets which affect tumor progression. Consequently, a better understanding of the TOX in glioma may be significant in the development of novel treatment strategies.

Based on an integrative and large-scale bioinformatic analysis, we delineated the clinical and molecular landscape of TOX among gliomas. TOX was found to be highly elevated in gliomas based on its mRNA expression levels, especially in LGG. TOX was up-regulated in MGMT promotor methylated glioma, glioma with IDH mutation, and glioma with 1p/19q codeletion. In addition, women tended to have a higher expression level of TOX. TOX^high^ was closely related with the CL and MES molecular subtypes, which made it a sensitive diagnostic marker for gliomas. TOX was localized to Cellular tumour, leading Edge, and pseudopalisading cells around necrosis in the IVY database. Moreover, high expression of TOX was associated with better survival in pan-glioma analysis, LGG alone, and GBM alone. We also explored the expression level of TOX with regard to distinct genomic alternations. We found that multiple somatic mutations had negative association with TOX expression, which suggested that TOX expression was irrelevant to the malignant biological process. All these results indicate that TOX expression occures in the wake of the glioma, and that TOX is critical in suppressing the oncogenic process and progression in this context. Oncogenic drivers including PIK3C2B, EGFR, and CDK4 are amplified in gliomas with low TOX expression [37]. Meanwhile, tumor suppressor genes, CDKN2A and PTEN, are deleted in cases with low TOX expression [38]. Given that genomic alternations may promote the progression of tumor through transforming the tumor microenvironment [39], these results suggest that TOX expression is associated with benign biological processes.

GBM elicits the activation of multiple immune cell types. While GBM has also been proven to rely on tumor infiltrating macrophages which produce numerous cytokines, growth factors, and interleukins that create a permissive tumor microenvironment, promoting glioma cell growth and proliferation [40]. In our study, TOX was found to negatively associate with macrophages, suppressing the permissive tumor microenvironment of GBM. Furthermore, correlation analysis suggested that TOX^high^ GBM cells are inclined to reject the infiltration of immune cells (cytotoxic lymphocytes, neutrophils, monocytic lineage, NK cells, B cells, and T cells) into the tumour microenvironment. These data suggest that TOX contributes to the anti-tumour immunity in the GBM microenvironment. Accumulating evidence has proven that TOX1 is critical in the generation and development of CD4 T cells [41], NK cells, and NKT cells [42, 43]. Therefore, our results are consistent with previous studies. Furthermore, pan-glioma analysis indicates that the negative correlation between TOX and immune infiltrating cells is much more significant in LGG samples than in GBM samples.

Previous studies have proven that APCs can present antigens to T cells in the central nervous system (CNS), which activated tumor-specific T cells (TST) can subsequently respond to in CNS tumors. Additionally, tumour progression influences the integrity of the blood brain barrier (BBB), which further enables a direct explosion of GBM to immune system [44]. TOX, regulating the differentiation of TST cells, is critical for the exhaustion of CD8 T cells by translating continuous stimulation into a distinct exhausted T (Tex) cell epigenetic and transcriptional developmental program [41, 45], preventing the overstimulation of T cells and subsequent activation-induced cell death under the stimulation of chronic antigen such as those present in cancer [46]. In our study, activated CD8 T cells were negatively associated with the TOX expression, which is also consistent with the previously reported function of TOX.

**Fig. 9:**
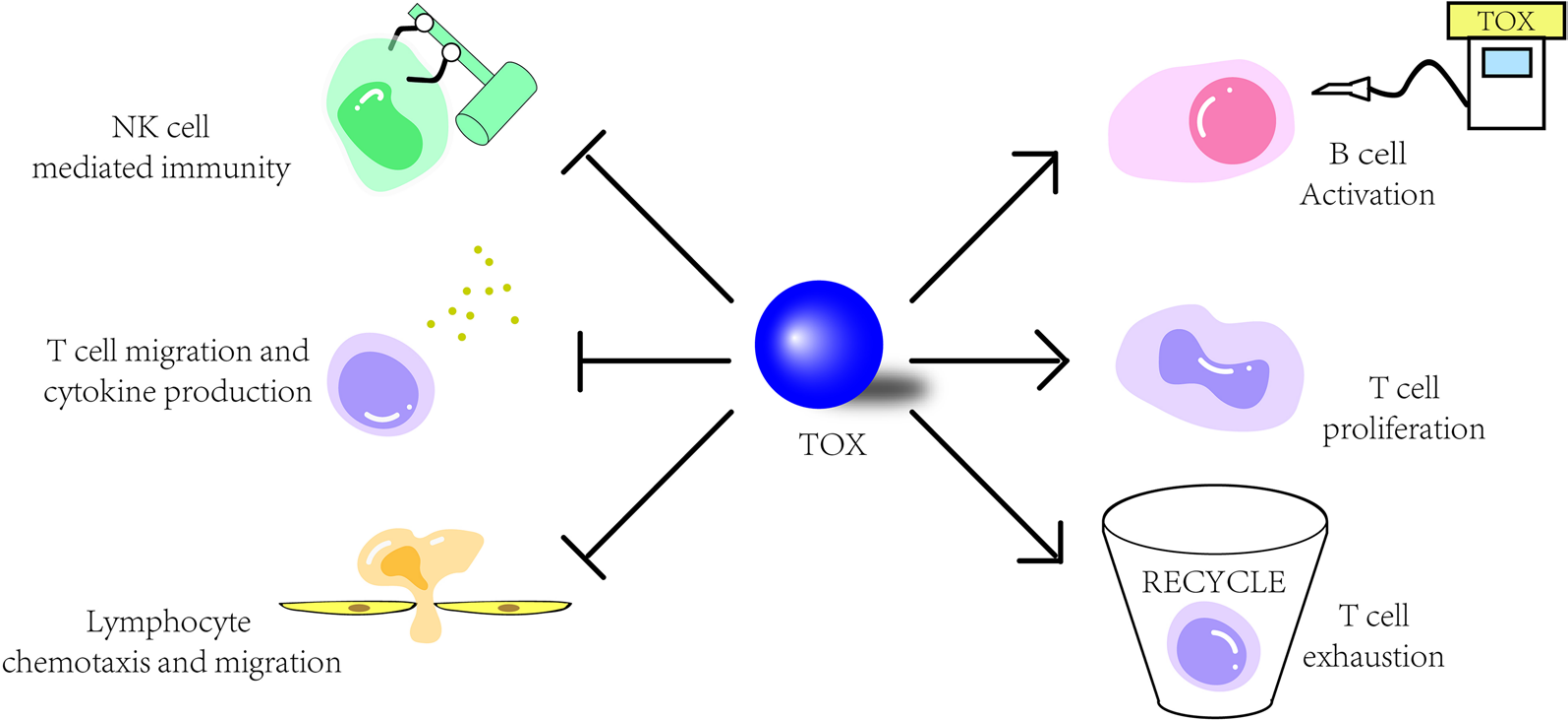
Working model of the effect of TOX in the immune system of glioma. TOX is highly expressed in glioma, regulating B cell activation and suppressing NK cell mediated immunity and lymphocyte chemotaxis and migration. TOX also mediates T cell proliferation and T cell exhaustion, while suppressing T cell migration and cytokine production

Through GSVA analysis, we revealed that TOX function was positively associated with immune related pathways including T cell receptor signaling pathway, T cell proliferation, and B cell activation, while negatively associated with lymphocyte migration, natural killer cell activation, and lymphocyte chemotaxis. These results suggest that TOX is correlated with the development and differentiation of B cells and T cells, and suppression of lymphocytes and natural killer cells in GBM. In addition, the pan-glioma analysis indicated that TOX had a negative association with T cell migration, negative T cell selection, regulation of T cell cytokine production, natural killer cell mediated immunity, positive regulation of T cell apoptotic process, B cell mediated immunity, lymphocyte migration, and lymphocyte chemotaxis, which further confirmed the lymphocyte-suppressing role of TOX in LGGs. Notably, the negative relation with lymphocyte migration and lymphocyte chemotaxis indicates that TOX is inclined to prohibit the formation of an immune infiltrating environment conducive to glioma. The interaction between TOX and immune system is illustrated in Fig. 9.

Preclinical benefits are seen with several immune checkpoint inhibitor treatments. Therefore, we also investigated the correlation between TOX and other immune checkpoint members. TOX had high correlation with CD276, IDO1, PDCD1LG2, and VTCN1 in both pan-glioma analysis and GBM alone. These results suggest that targeting TOX and other immune checkpoint molecules could be a novel approach to treat gliomas.

## Conclusion

Taken together, this study illuminates the role that TOX plays in the development of human gliomas. Notably, TOX seems to have a more significant correlation with LGG than with GBM. Future studies are warranted to explore TOX as a new prognostic marker or immune-therapeutic mediator for GBMs and LGGs, and subsequent pharmaceutical research in regard to TOX may demonstrate promising results.

## Supplementary information

**Additional file 1: Fig. S1.** TOX expression is associated with better survival in glioma patients. Kaplan-Meier analysis of overall survival (OS) based on high vs low expression of TOX in pan-glioma analysis, and LGG and GBM patients in CGGA dataset. The median value of TOX expression was used as the cut-off value. P-values were obtained from the log-rank test. Heatmaps illustrating TOX related inflammatory activities in GBM (A) and pan-glioma analysis (B) in TCGA dataset, respectively. Expression values are z-transformed and are colored red for high expression and blue for low expression, as indicated in the scale bar. Correlation-grams illustrate P values for analysis between TOX and inflammatory metagenes in GBM (C) and pan-glioma analysis (D) in TCGA dataset, respectively.

**Additional file 2: Fig. S2.** TOX is associated with immune cell populations in the tumour microenvironment. Correlation-grams illustrate P values for analysis between TOX and 28 immune cells in TCGA GBM (A), LGG (C) and pan-glioma analysis data (E), respectively. Correlation-grams illustrate P values for analysis between TOX and 24 immune cells in TCGA GBM (B), LGG (D)and pan-glioma analysis data (F), respectively.

**Additional file 3: Fig. S3.** TOX is associated with immune cell populations in the tumour microenvironment. Heatmaps illustrating the relationship between TOX and 24 immune cell populations based on CGGA pan-glioma analysis (A) and GBM (B), respectively. Heatmaps illustrating the relationship between TOX and 28 immune cell populations based on CGGA pan-glioma analysis (C) and GBM (D), respectively. Expression values are z-transformed and are colored red for high expression and blue for low expression, as indicated in the scale bar. Correlation-grams illustrate P values for analysis between TOX and 24 immune cells in CGGA pan-glioma analysis (E) and GBM (F), respectively. Correlation-grams illustrate P values for analysis between TOX and 28 immune cells in CGGA pan-glioma analysis (G) and GBM (H), respectively.

**Additional file 4: Fig. S4.** TOX is associated with other immune checkpoint molecules in gliomas. Correlation of TOX and immune checkpoint molecules in pan-glioma analysis (A), GBM (B) and LGG (C) samples in CGGA. Correlation of TOX and other family members in pan-glioma analysis (D), GBM (E) and LGG (F) samples in CGGA.

## Data Availability

The datasets generated and analyzed during the current study are available in the Gene Expression Omnibus (https://www.ncbi.nlm.nih.gov/geo/), TCGA data source (https://xena.ucsc.edu) and CGGA data portal (http://www.cgga.org.cn).

## Data availability sharing

Data sharing is not applicable to this article as no new data were created or analyzed in this study.
