# Correction to: Clinical characterization, genetic profiling, and immune infiltration of TOX in diffuse gliomas

**DOI:** 10.1186/s12967-020-02514-6

**Published:** 2020-09-28

**Authors:** Hao Zhang, Fan Fan, Yuanqiang Yu, Zeyu Wang, Fangkun Liu, Ziyu Dai, Liyang Zhang, Zhixiong Liu, Quan Cheng

**Affiliations:** 1 Department of Neurosurgery, Xiangya Hospital, Central South University, Changsha, 410008 Hunan People’s Republic of China; 2 Department of Clinical Pharmacology, Xiangya Hospital, Central South University, Changsha, 410008 Hunan People’s Republic of China; 3 Center for Medical Genetics and Hunan Provincial Key Laboratory of Medical Genetics, School of Life Sciences, Central South University, Changsha, China; 4 National Clinical Research Center for Geriatric Disorders, Xiangya Hospital, Central South University, Changsha, Hunan China; 5 Department of Medicine, The University of Oklahoma Health Sciences Center, Oklahoma City, OK 73104 USA; 6 Clinical Diagnosis and Therapeutic Center of Glioma, Xiangya Hospital, Central South University, Changsha, 410078 Hunan People’s Republic of China

## Correction to: J Transl Med (2020) 18:305 10.1186/s12967-020-02460-3

Following publication of the original article [1], the authors identified an error in Fig. 2f. The IHC image of GBM was mistakenly used for LGG. The correct Fig. 2 (as part of the complete Fig. 2) is given below. The authors also identified errors in Fig. 3. The ‘annotations’ for all of the parts were provided incorrectly. The correct Fig. 3 is given below.

**Fig. 2:**
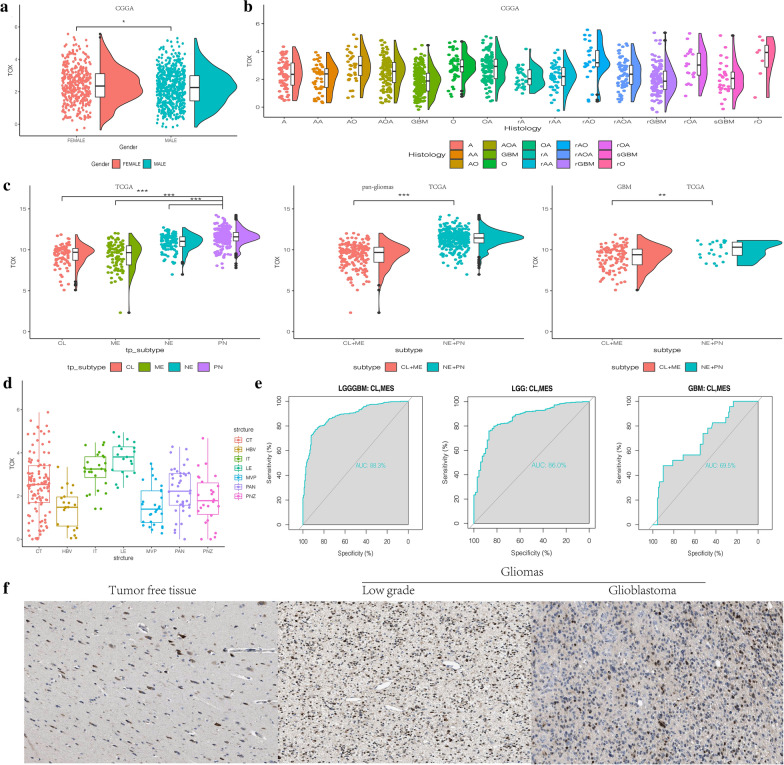
**a** TOX expression is upregulated in female patients with gliomas from CGGA. **b** The expression levels of TOX based on the histopathologic classification from CGGA. A, low-grade astrocytoma; AA, anaplastic astrocytoma; AO, anaplastic oligodendroglioma; GBM, glioblastoma; O, oligodendroglioma; rA, recurrent low-grade astrocytoma; rAA, recurrent anaplastic astrocytoma; rGBM, recurrent glioblastoma; rO, recurrent oligodendroglioma; sGBM, sensitive glioblastoma; AOA, anaplastic oligoastrocytoma; OA, oligoastrocytoma. **c** The TOX expression pattern in the TCGA molecular subtype in pan-glioma analysis and GBM samples. **d** TOX expression is detected in different anatomic locations for GBM in the IVY GBM database. LE (Leading Edge), IT (Infiltrating Tumour), CT (Cellular Tumour), PAN (Pseudopalisading Cells Around Necrosis), PNZ (Perinecrotic Zone), MVP (Microvascular Proliferation), and HBV (Hyperplastic Blood Vessels). **e** ROC curves predict that TOX is a biomarker of classical and mesenchymal subtype glioma. **f** TOX is more highly expressed in LGG than in GBM at the protein level

**Fig. 3:**
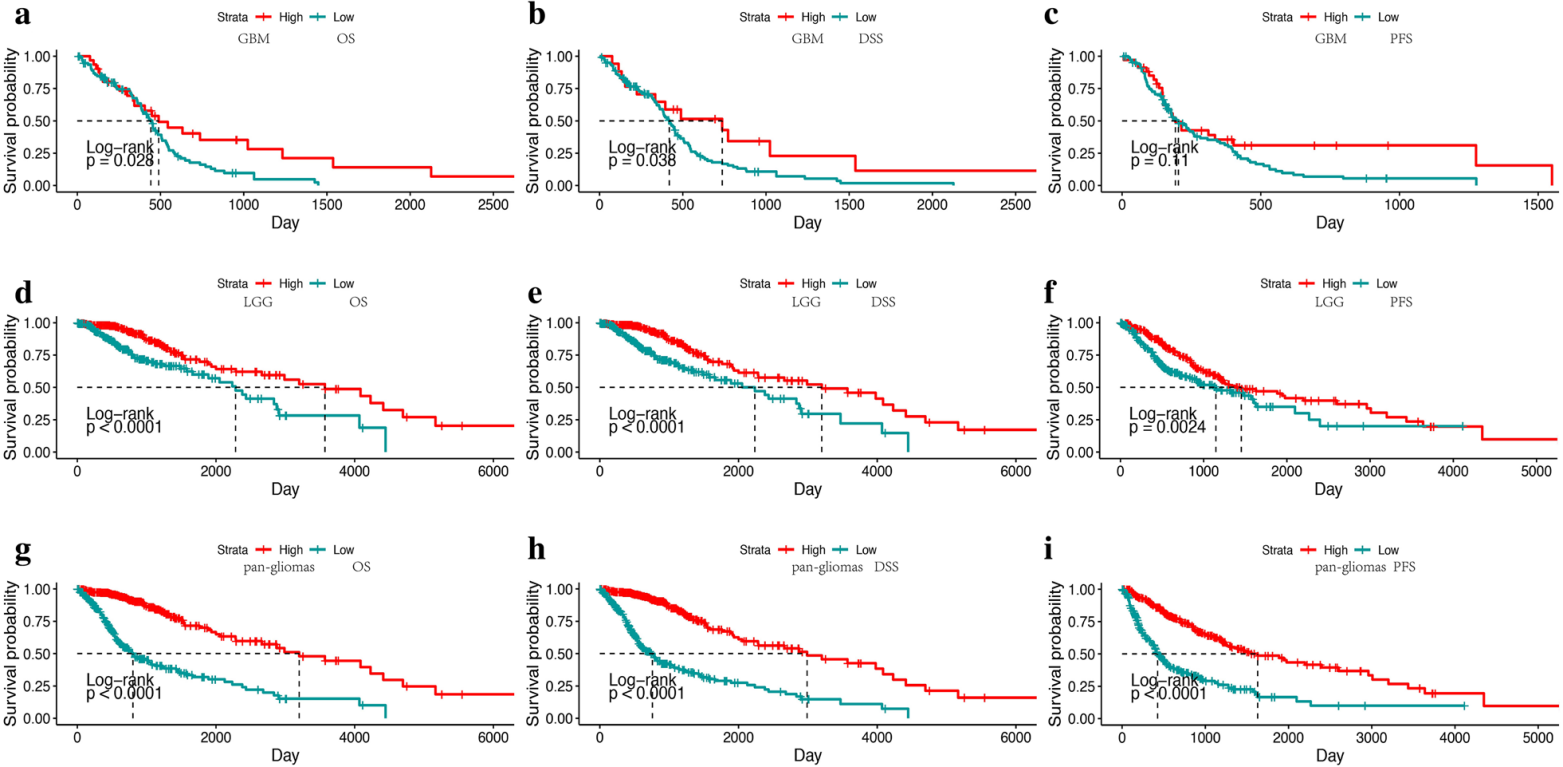
TOX expression predicts better survival in glioma patients. Kaplan–Meier analysis of overall survival (OS), disease specific survival (DSS) and progressive free survival (PFS) based on high vs low expression of TOX in pan-glioma analysis, LGG alone, and GBM alone in the TCGA dataset. The median value of TOX expression was used as the cut-off value. P-values were obtained from the log-rank test

Additionally, the authors identified the following errors in the main text and figure captions:

In the ‘TOX is irrelevant to inflammatory activities’ section, the text “but positively associated with the IgG metagene in panglioma analysis, LGG alone, and GBM alone (Fig. 6a–c; Additional file 1: S1D–G).” was corrected to “but positively associated with the IgG metagene in GBM alone, LGG alone, and pan-glioma analysis (Fig. 6a–c; Additional file 1: S1D–G).”

In the caption for Fig. 5, the sentence “TOX related immune processes in pan-glioma analysis (**a**), LGG (**b**) and GBM (**c**) patients in the TCGA dataset.” was corrected to “TOX related immune processes in pan-glioma analysis (**a**), GBM (**b**) and LGG (**c**) patients in the TCGA dataset.”

The caption for Fig. 6 was originally provided as “Heatmaps illustrating TOX related inflammatory activities in GBM (**a**) and pan-glioma analysis (**b**) in TCGA dataset, respectively. Expression values are z-transformed and are colored red for high expression and blue for low expression, as indicated in the scale bar. Correlation-grams illustrate P values for analysis between TOX and inflammatory metagenes in GBM (**c**) and pan-glioma analysis (**d**) in TCGA dataset, respectively.” The caption was corrected to “Heatmaps illustrating TOX related inflammatory activities in GBM (**a**) and pan-glioma analysis (**b**) in CGGA dataset, respectively. Expression values are z-transformed and are colored red for high expression and blue for low expression, as indicated in the scale bar. Correlation-grams illustrate P values for analysis between TOX and inflammatory metagenes in GBM (**c**) and pan-glioma analysis (**d**) in CGGA dataset, respectively.”

The original article has been corrected.
