# Supplementary figures and images for: Clinical characterization, genetic profiling, and immune infiltration of TOX in diffuse gliomas
Source: J Transl Med. 2020 Aug 6;18:305. doi: 10.1186/s12967-020-02460-3 (PMC7409670; doi:10.1186/s12967-020-02460-3)

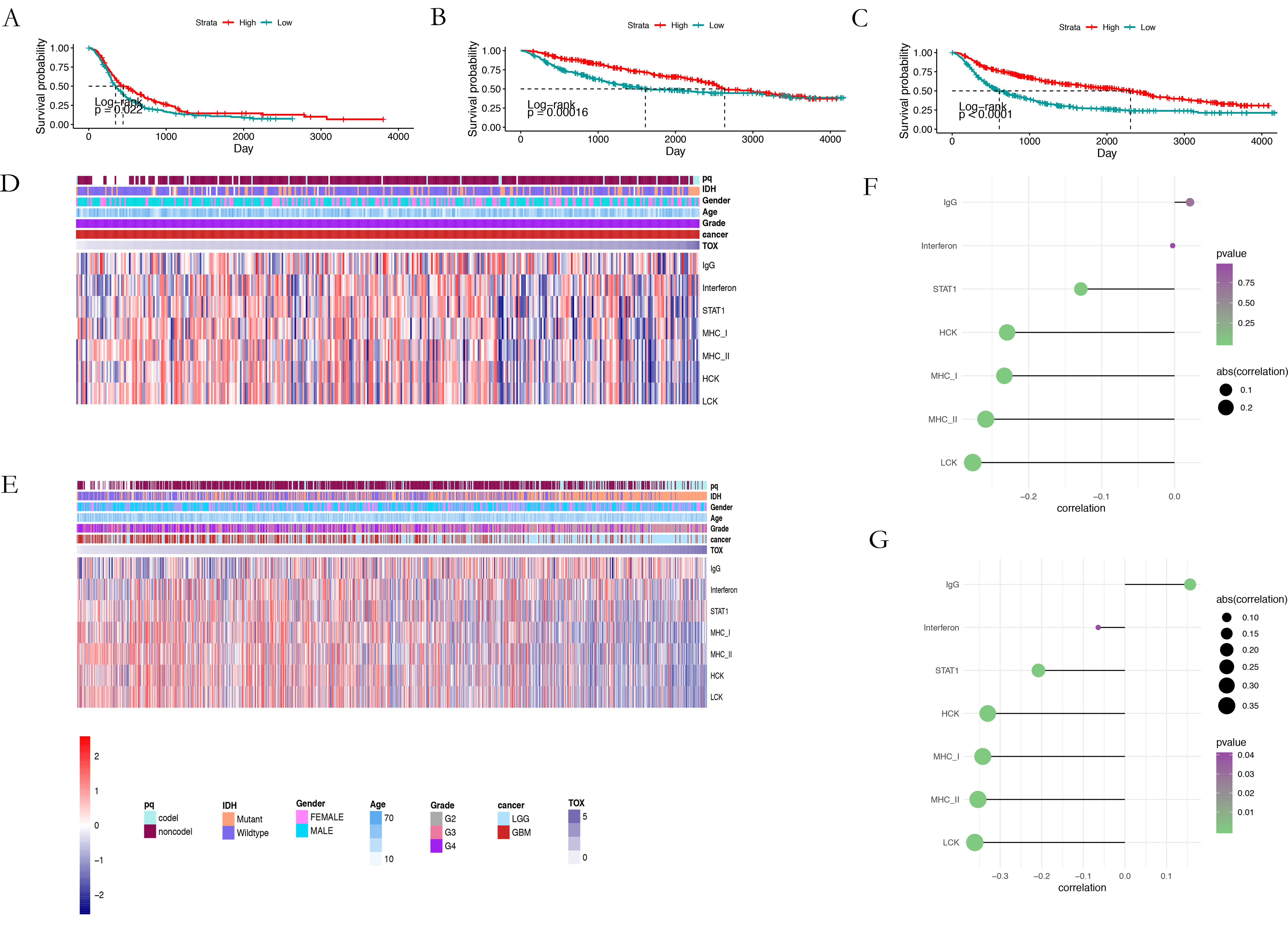

Supplement: Supplementary file 1 — Additional file 1: Fig. S1. TOX expression is associated with better survival in glioma patients. Kaplan-Meier analysis of overall survival (OS) based on high vs low expression of TOX in pan-glioma analysis, and LGG and GBM patients in CGGA dataset. The median value of TOX expression was used as the cut-off value. P-values were obtained from the log-rank test. Heatmaps illustrating TOX related inflammatory activities in GBM (A) and pan-glioma analysis (B) in TCGA dataset, respectively. Expression values are z-transformed and are colored red for high expression and blue for low expression, as indicated in the scale bar. Correlation-grams illustrate P values for analysis between TOX and inflammatory metagenes in GBM (C) and pan-glioma analysis (D) in TCGA dataset, respectively. [file 12967_2020_2460_MOESM1_ESM.jpg]

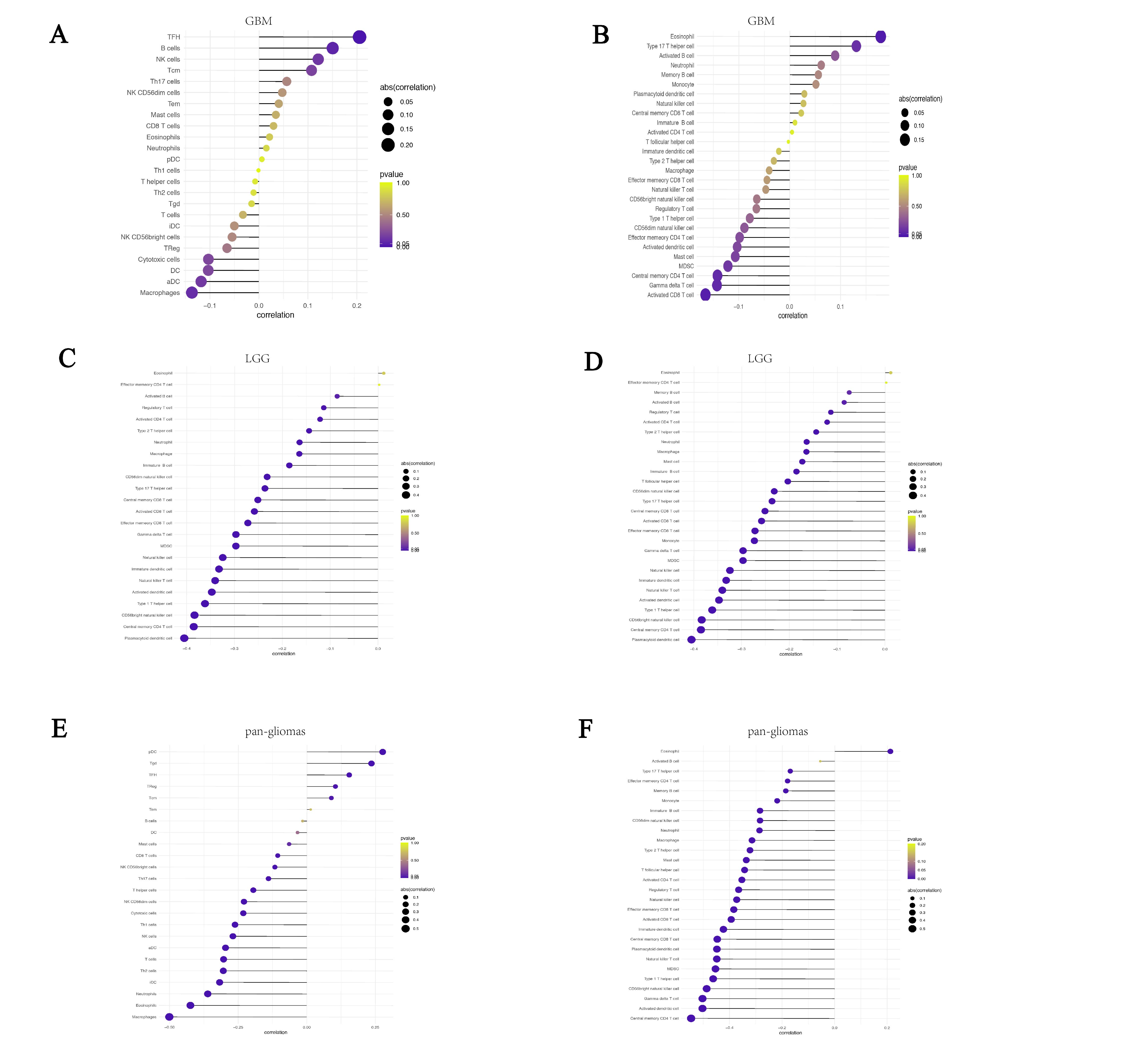

Supplement: Supplementary file 2 — Additional file 2: Fig. S2. TOX is associated with immune cell populations in the tumour microenvironment. Correlation-grams illustrate P values for analysis between TOX and 28 immune cells in TCGA GBM (A), LGG (C) and pan-glioma analysis data (E), respectively. Correlation-grams illustrate P values for analysis between TOX and 24 immune cells in TCGA GBM (B), LGG (D)and pan-glioma analysis data (F), respectively. [file 12967_2020_2460_MOESM2_ESM.jpg]

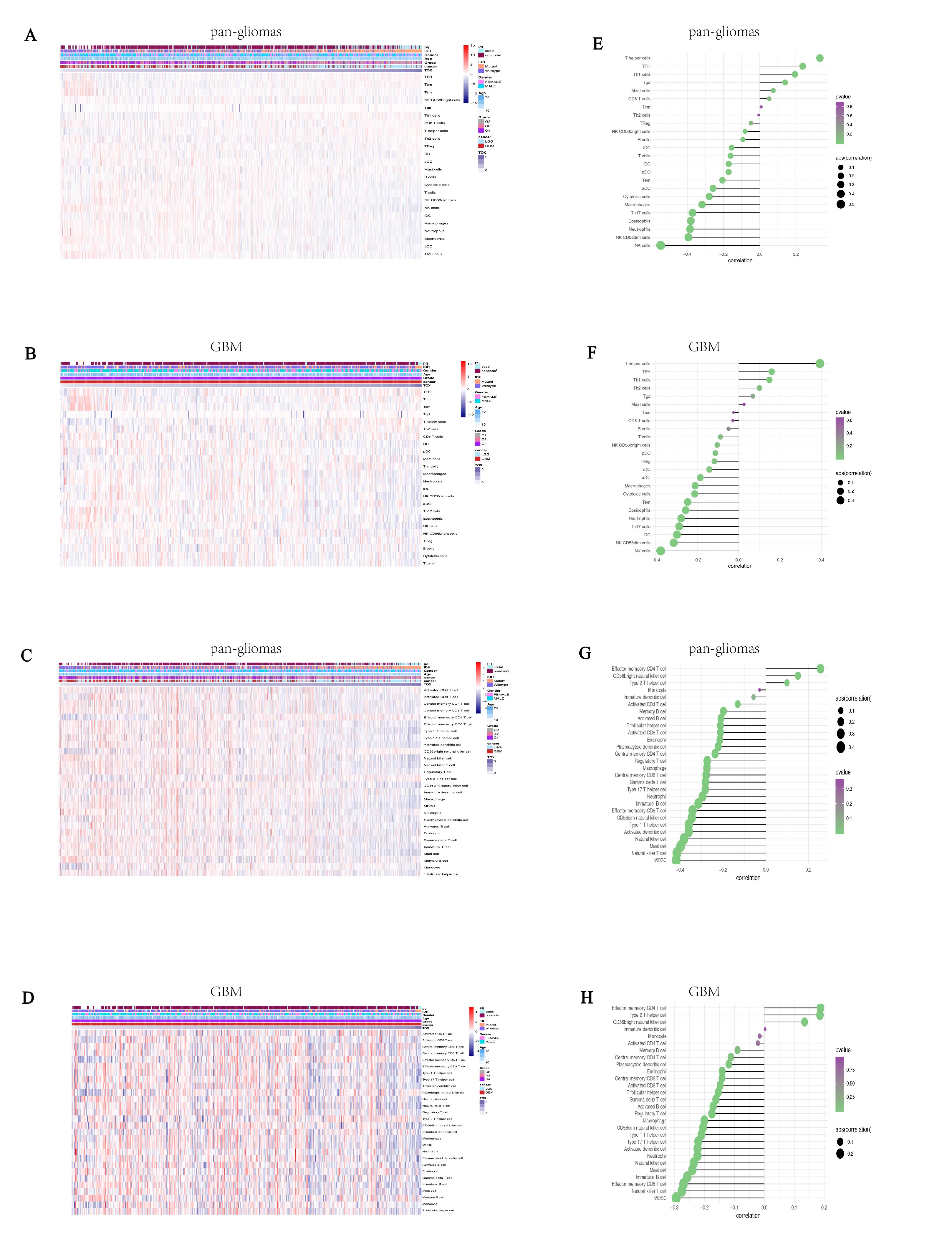

Supplement: Supplementary file 3 — Additional file 3: Fig. S3. TOX is associated with immune cell populations in the tumour microenvironment. Heatmaps illustrating the relationship between TOX and 24 immune cell populations based on CGGA pan-glioma analysis (A) and GBM (B), respectively. Heatmaps illustrating the relationship between TOX and 28 immune cell populations based on CGGA pan-glioma analysis (C) and GBM (D), respectively. Expression values are z-transformed and are colored red for high expression and blue for low expression, as indicated in the scale bar. Correlation-grams illustrate P values for analysis between TOX and 24 immune cells in CGGA pan-glioma analysis (E) and GBM (F), respectively. Correlation-grams illustrate P values for analysis between TOX and 28 immune cells in CGGA pan-glioma analysis (G) and GBM (H), respectively. [file 12967_2020_2460_MOESM3_ESM.jpg]

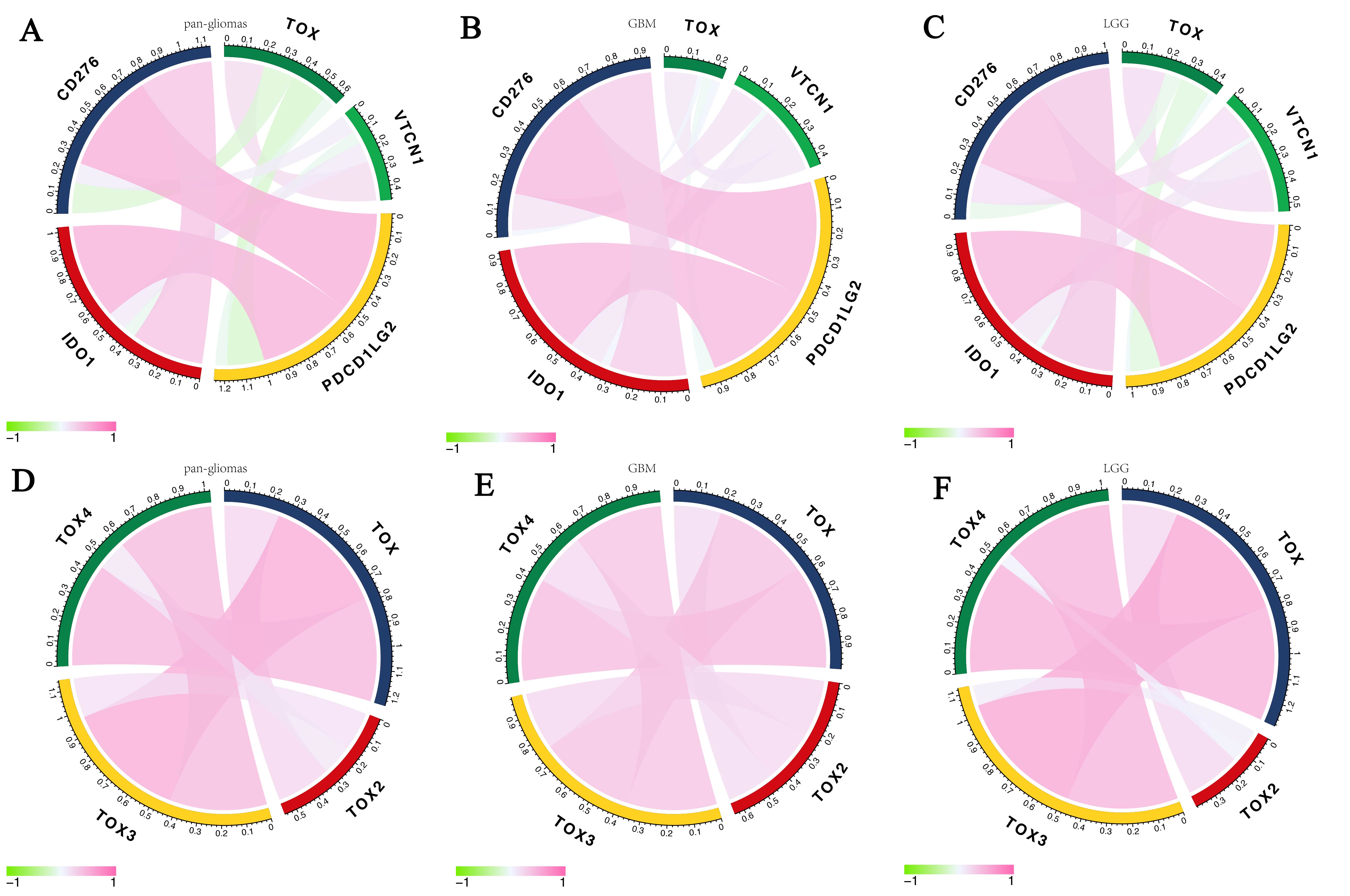

Supplement: Supplementary file 4 — Additional file 4: Fig. S4. TOX is associated with other immune checkpoint molecules in gliomas. Correlation of TOX and immune checkpoint molecules in pan-glioma analysis (A), GBM (B) and LGG (C) samples in CGGA. Correlation of TOX and other family members in pan-glioma analysis (D), GBM (E) and LGG (F) samples in CGGA. [file 12967_2020_2460_MOESM4_ESM.jpg]
